# Autoethnography helps analyse emotions

**DOI:** 10.3389/fpsyg.2015.00209

**Published:** 2015-02-25

**Authors:** Ralf Buckley

**Affiliations:** International Chair in Ecotourism Research, School of Environment, Griffith UniversityGold Coast, QLD, Australia

**Keywords:** risk, fear, thrill, recreation, emergencies, methods and platform development, time perception

Emotions are a near-universal component of human experience, with powerful influences on human attitudes and behaviors, and large-scale social, economic, and environmental consequences (Zajonc, 1984; Oatley et al., 2006; Turner and Stets, 2006; Nesse and Ellsworth, 2009; Niedenthal and Brauer, 2012; Dehaene, 2014). They have been described as a continuum that includes over 100 named emotions, with some individual and cultural differences (Johnson-Laird and Oatley, 1989; Nesse and Ellsworth, 2009; Izard, 2010). These are sometimes treated as combinations of basic emotions (Ortony and Turner, 1990), recognizable through facial expressions (Ekman, 1982; Dannlowski et al., 2007; Graham and LaBar, 2012).

The neurophysiological and biochemical mechanisms and effects of human emotions have been analyzed intensively (LeDoux, 2000; Ackerl et al., 2002; Chen et al., 2006; Mujica-Parodi et al., 2009; Zhou and Chen, 2009; Haegler et al., 2010; Van Westerloo et al., 2011; Agren et al., 2012; Gross and Canteras, 2012; Avila and Lin, 2014; Dehaene, 2014). All such measures, however, rely on research subjects also communicating their self-perceived emotions to the researcher. Similarly, for analyses of psychological experiences rather than biochemical or neurological mechanisms, research subjects must express their emotional experiences in words and communicate them to researchers. The latter also use language to communicate their analyses to research readers. They may include selected quotations directly from the research subjects. Comprehension of such communications about emotions depends on mutual recognition of similar emotional experiences.

This approach breaks down, however, for emotions that are not widely experienced, and which are considered to be indescribable by those who have indeed experienced them. The popular literatures of ecstatic religions, active military combat, and extreme outdoor sports all argue that there are feelings that are only comprehensible to individuals who have experienced them in person (Caputo, 1977; Allman et al., 2009; Buckley, 2012; Yogis, 2013). These experiences include the perception of slowed time (Arstila, 2012; Wittmann, 2013; Buckley, 2014).

Human societies thus include a small number of individuals who have experienced emotions of a type or intensity that fall outside the distributional range for the remainder of the population. These differences are recognized by the rest of society, which may label those individuals as eccentric, sick or crazy, with either positive or negative connotations. For at least some individuals, these emotional experiences drive them to take actions that are considered unusual or extreme, also in either a negative or positive way. There are extensive literatures on the psychologies of crime, combat, mental illness, spirituality and extreme sport, reflecting the importance of these emotions and actions to human societies (Lyng, 1990; Buckley, 2012).

These literatures include ethnographic analyses, where the researcher aims to become assimilated into the group under study. Even with full-immersion ethnography, however (Hammersley and Atkinson, 2007; Dauphinee, 2010), verbal communication between research subject and research analyst remains critical. The insider approach aims partly to provide a shared basis for understanding, but principally to establish trust between subjects and researcher, so that the research subjects will tell the researcher information that they do not share with outsiders. Even insider ethnographies, therefore, cannot be used to analyse experiences which research subjects cannot put into words. If a research subject cannot communicate their emotional experiences to a researcher, there is no opportunity for analysis unless the researcher can directly experience those same emotions themselves.

For emotional experiences of this type, therefore, the only research approach currently available is analytical autoethnography, the systematic study of a researcher's own experience (Buzard, 2003; Anderson, 2006; Dauphinee, 2010; Tolich, 2010). Researchers who have themselves undergone such experiences have an opportunity to describe, analyse and report them by using themselves as research subjects. Extreme emotional experiences are infrequent, and most are involuntary (Berntsen, 2009), so data are sparse. In the particular case of extreme sports or adventure activities, however, a researcher who is also a practitioner can adopt an experimental approach to autoethnography. That is, they can deliberately repeat an experience which creates powerful emotions, specifically to study those emotions.

Analytical autoethnography may thus be seen simply as an extension or special case of conventional ethnography. In any ethnographic study, there is a trade-off between breadth and depth, the number of research subjects against the detail they reveal. Analytical autoethnography is the logical continuation, narrowest but deepest. Researchers can examine their own emotions in finer detail than those of research subjects. Using a retrospective approach, they can replay past experiences repeatedly from memory. Using a prospective or experimental approach, they can deliberately replicate particular experiences to analyse the associated emotions.

The practical methodology for retrospective analytical autoethnography of intense human emotional experiences is the same as for conventional ethnography (Hammersley and Atkinson, 2007). In each case, data are derived from detailed recollections (Berntsen, 2009; Rubin, 2014) of brief, infrequent but critical (Flanagan, 1954) lifetime events. These events are fixed firmly in memory precisely because of the intensity of associated emotions (Levine and Pizarro, 2004; Rubin, 2014). The research focus is on self-perceived emotional experiences as they exist in the subject's memory. These recollections thus provide precisely the data required.

Some examples of such data are provided by Buckley (2012). Other examples, from the author's own autoethnographic experience, include the details of thrill, joy, triumph, fear, relief, anger and sadness experienced during events such as narrowly escaping death, seeing friends die, commencing difficult maneuvres during outdoor adventure sports, and either completing such maneuvres successfully, or failing but surviving. Most of these critical events occurred during adventure recreation activities. Other researchers have described their own experiences in very different circumstances, such as combat, religion or medicine.

In recalling intense emotional experiences, analytical autoethnographers can use three measures of intensity. In some cases, intense emotions triggered involuntary actions or vocalizations at the time they were experienced, and these can be recalled later. Examples include gasps of awe or yells of triumph (Buckley, 2012). In other cases, the recollection includes particularly fine and intense details of sensory perceptions unrelated to the emotion itself. In other cases again, the recollection generates an involuntary physiological response, even if the actual event was decades early (Mattley, 2002; Levine and Pizarro, 2004). An example is a cold sweat on recalling a narrow escape (Buckley, 2012). In the experimental approach, an autoethnographer can learn through practice to examine the fine-scale temporal sequences of actions and emotions, at a resolution rarely available through retrospective evaluation of involuntary experiences.

As with all forms of ethnography, triangulation is a critical component, to provide reliability and confidence in individual recollections. Readers of any ethnographic research rely on research subjects to report their experiences reliably and accurately to the ethnographers, and ethnographers to select and report their subjects' statements accurately to readers. Both analyst and readers triangulate information from individual research subjects against other relevant sources, to test reliability. Neither have direct access to the subjects' own emotional experiences. The same applies to autoethnographies. As in all forms of research, research readers rely on researchers' abilities to make accurate and undeluded observations. If a researcher is able to undergo and describe an experience reported by others as real but indescribable, then their descriptions can be triangulated against ethnographies and autobiographies, adding reliably to understanding of intense human emotions.

Historically, it appears that there has been a substantial bias against analytical autoethnography in many social sciences. This, however, appears to be unjustified. Autoethnographic observations have the advantage of being first-hand, unlike the second-hand reports from conventional ethnographies. The natural sciences routinely rely on first-hand rather than second-hand observations. A number of famous scientific discoveries have been made by researchers using themselves as experimental subjects (Moult, 2007). In legal testimony, a witness's own first-hand observations are admissible evidence, whereas second-hand hearsay is not. In some social sciences, autoethnographic approaches have yielded significant new insights (Holyfield, 1999; Buckley, 2012; Houge Mackenzie and Kerr, 2013). I suggest that with appropriate precautions, analytical autoethnography can also make unique contributions in psychology. There is no reason to exclude a valid and valuable source of data, especially where it can reveal information not otherwise available.

There is also a further possibility to extend conventional ethnography into a more experimental approach, using analytical autoethnography as a stepping stone. There are some experiences which research subjects in conventional ethnographies are unable to describe, but which suitably experienced analytical autoethnographers can indeed describe and analyse. Those single-subject analyses could then be used as hypotheses against which to test the recollections of ethnographic subjects, through repeated interviews. The prospective approach to analytical autoethnography could also be extended to conventional ethnography, by training research subjects to observe their own emotions during particular actions and experiences.

Both these approaches would require ethnographers to renegotiate their relationships with their research subjects. For topics such as crime or espionage where the researcher must remain disguised, this may not be possible. For topics such as extreme adventure sports, however, it is entirely feasible. The first step, however, is to recognize that through analytical autoethnography, the direct experiences of a researcher are not merely a tool in elucidating the experiences of others, but valid data in themselves.

## Conflict of interest statement

The author declares that the research was conducted in the absence of any commercial or financial relationships that could be construed as a potential conflict of interest.
